# Evaluation of Hs-CRP Levels and Interleukin 18 (-137G/C) Promoter Polymorphism in Risk Prediction of Coronary Artery Disease in First Degree Relatives

**DOI:** 10.1371/journal.pone.0120359

**Published:** 2015-03-30

**Authors:** Rajesh Kumar G, Mrudula Spurthi K, Kishore Kumar G, Mohanalatha Kurapati, Saraswati M, Mohini Aiyengar T, Chiranjeevi P, Srilatha Reddy G, Nivas S, Kaushik P, Sanjib Sahu K, Surekha Rani H

**Affiliations:** 1 Department of Genetics, Osmania University, Hyderabad 500007, Telangana, India; 2 Bioclues.org, Kukatpally, Hyderabad 500072, Telangana, India; 3 Durgabai Deshmukh Hospital and Research Center, Vidyanagar, Hyderabad 500007, Telangana, India; Morehouse School of Medicine, UNITED STATES

## Abstract

**Background:**

Coronary Artery Disease (CAD) is clearly a multifactorial disease that develops from childhood and ultimately leads to death. Several reports revealed having a First Degree Relatives (FDRS) with premature CAD is a significant autonomous risk factor for CAD development. C - reactive protein (CRP) is a member of the pentraxin family and is the most widely studied proinflammatory biomarker. IL-18 is a pleiotrophic and proinflammatory cytokine which is produced mainly by macrophages and plays an important role in the inflammatory cascade.

**Methods and Results:**

Hs-CRP levels were estimated by ELISA and Genotyping of IL-18 gene variant located on promoter -137 (G/C) by Allele specific PCR in blood samples of 300 CAD patients and 300 controls and 100 FDRS. Promoter Binding sites and Protein interacting partners were identified by Alibaba 2.1 and Genemania online tools respectively. Hs-CRP levels were significantly high in CAD patients followed by FDRS when compared to controls. In IL-18 -137 (G/C) polymorphism homozygous GG is significantly associated with occurrence of CAD and Hs-CRP levels were significantly higher in GG genotype subjects when compared to GC and CC. IL-18 was found to be interacting with 100 protein interactants.

**Conclusion:**

Our results indicate that Hs-CRP levels and IL-18-137(G/C) polymorphism may help to identify risk of future events of CAD in asymptomatic healthy FDRS.

## Introduction

Coronary artery disease (CAD) is one of the major health problems worldwide. The total burden of coronary artery disease is predicted to increase substantially over the next few years as a dreadful rise in the global burden of CAD for the past 15 years has been put forth by several epidemiologists in India and worldwide such as WHO, Global burden of disease study etc. Atherosclerosis a principal cause of CAD is a consequence of multiple factors that lead to the deposition of athermatous plaques in and around the coronary artery walls that supply oxygen and nutrients to the myocardium. The atherosclerotic clinical symptoms include Unstable Angina (UA), Myocardial Infarction (MI), stroke and sudden cardiac arrest [1, 2].

Multiple risk factors for atherosclerosis have been reported, that include hypertension, smoking, high LDL cholesterol levels, diabetes, obesity, physical inactivity, male sex, insulin resistance etc and are categorized mainly as genetic (20–60%) and environmental (50%) risk factors [3,4]. Certain risk factors are interdependent on both these factors [5].

Recent epidemiological studies have reported that a family history of CAD is an autonomous risk factor for CAD development. In particular, having a family history is associated with 1.5 to 2 fold increase in risk of developing cardiovascular disease. Furthermore, maternal family history of CAD may even predict future cardiovascular events more strongly than a paternal family history [6].

Inflammation is involved in almost all stages of atherosclerosis, right from initiation of lesion to successive progression and final plaque destabilization. In addition, inflammation regulates both the “solid-state” thrombotic potential in the plaque itself and the prothrombotic and anti-fibrinolytic capacity of blood in the fluid phase. The ominous presence of inflammation in atherosclerosis has prompted the evaluation of certain key inflammatory factors in cardiovascular risk prediction [7].

The best proved inflammatory biomarker recently is CRP; other markers include soluble CD40 ligand, adiponectin, interleukin 18, and matrix metalloproteinase 9, etc., also gives additional information for risk prediction of CAD [7].

C-reactive protein (CRP), the classic acute-phase protein, is not directly involved in the coagulation process but is an exquisitely sensitive objective marker of inflammation, tissue damage, and infection reflecting the degree of underlying inflammatory response and provides a useful measure of immune injury to tissues [8].

Interleukin-18 (IL-18) is a pleiotropic proinflammatory cytokine plays an important role in the inflammatory cascade. Evidence suggests that IL18 gene single nucleotide polymorphisms (SNPs) have been associated with the varying circulating levels of IL18 and may be related to atherosclerotic plaque progression and vulnerability. These SNPs have been reported to be associated with clinical outcome in subjects with CAD [9].

## Objective

The aim of the present study is to investigate the Hs-CRP levels, IL-18 polymorphisms and promoter binding sites & its protein-protein interaction to find out the underlying mechanisms and predisposition of disease in CAD patients and their first degree relatives (FDRS) in comparison with controls.

## Materials and Methods

The study population consisted of 300 patients with angiographically documented CAD, admitted at the cardiology unit of Durgabai Deshmukh Hospital and Research Center, Hyderabad and 100 asymptomatic FDRS of the patients and 300 healthy individuals with no known history of any disease, as controls. The healthy controls subjects representing same geographical location without any history of CAD were included after a thorough interview and clinical examination. Patients with concomitant valvular heart disease, cardiomayopathy, acute renal failure, acute and chronic viral or bacterial infections, asthama, tumours or connective tissue diseases and those who are on dialysis were excluded from the study.

This study was approved by Institutional Ethics Committee for Biomedical Research of Institute of Genetics & Hospital for Genetic Diseases, Hyderabad.

Detailed written informed consent was obtained from all the participants of this study. All the subjects were examined clinically and detailed history was recorded with particular reference to the known risk factors for CAD, including family history, hypertension, diabetes mellitus, smoking, food habits, life style etc.

Following an overnight fast, blood samples were drawn by vein puncture into two tubes, with and without anticoagulant for biochemical and molecular analysis. Estimation of lipid profiles, Hs-CRP and genotypic evaluation of IL-18 polymorphism was carried out in the samples of CAD, FDRS and controls.

### Estimation of Lipid Profiles

Estimation of Lipid Profiles was carried out using commercially available kits from Randox UK.

### Estimation of Hs CRP levels

Estimation of CRP levels was carried out using commercially available Enzyme Linked Immunosorbent Assay (ELISA) with a typical two-step capture assay kit from Diagnostic Biochem Canada Inc.

### Genotyping

Genomic DNA was isolated from fresh blood by salting out procedure [10] stored at -20°C. Interleukin 18-137G/C (rs187238) gene polymorphism was carried out by Allele specific step down PCR method. Briefly, two complementary reactions were established for each allele along with the common reverse primer (5’-AGGAGGGCAAAATGCACTGG-3’) and two allele specific primers (5’-CCCCAACTTTTACGGAAGAAAA**C**-3’ and 5’-CCCCAACTTTTACGG AAGAAAA**G**-3’) respectively and control forward primer (5’-CCAATAGGACTGATTATT CCGCA-3’) (internal positive amplification control).

Each amplification reaction contained 5 μl of 10X buffer containing 15 mM MgCl_2_, 0.5 μl of 10 pm of each primer, 0.25 μl of 10mM dNTPs (Merck), 0.2 U of Taq polymerase (Merck) and 2 μl of DNA.

Reactions were carried out in a Bio-Rad Thermo Cycler (Bio-Rad Laboratories, Hercules, CA, USA). Initial denaturation at 94°C for 2 minutes, denaturation at 94°C for 20 seconds, annealing at 64.5°C for 40 seconds and extension at 72°C for 70 seconds as the first step followed by 25 cycles of denaturation at 94°C for 20 seconds, annealing at 61°C for 40 seconds and extension at 72°C for 40 seconds. The final extension was performed at 72°C for 5 minutes.

All PCR products were visualized under UV light on ethidium bromide-stained 2% agarose gels. An amplification product of 261 bp was detected for G & C and 446 bp for control forward primer.

### Promoter Binding Site

Promoter binding sites for IL-18 -137G>C polymorphism have been determined by using Alibaba 2.1 online bioinformatics tool.

### Protein-Protein Interaction Analysis

To understand the kind of networking and the genes linked with IL 18 in protein-protein interaction studies were performed *in silico* using Genemania web server <http://genemania.org/>.

IL18 was queried under the human grid for the closest 100 interactants.

### Statistical analysis

Statistical analysis for our data was performed using version 9, (SAS Institute Inc, Cary, NC, USA). Continuous clinical data was compared by unpaired Student’s *t-*test and presented as mean ± standard deviation (SD). The χ2 test was used to compare discrete variables and to compare genotype distributions.

The Hardy-Weinberg equilibrium was confirmed using the χ2 test. A *p* value of <0.05 was considered to be statistically significant. A two-tailed probability test of 0.05 or less was considered statistically significant. Promoter Binding sites and Protein interacting partners were identified by Alibaba 2.1 and Genemania online tools respectively.

## Results

The number of patients, FDRS and controls along with their clinical and demographic data is shown in table 1. The risk factors for coronary artery disease including age, gender, smoking (male), non-vegetarians, family history and lipid profiles were found to be significant in CAD patients followed by FDRS compared to controls.

**Table 1:** Clinical and Demographic data of subjects.

	Controls	First degree relatives	Coronary artery disease
**Age(years)**	52.01±8.08	42.60±15.3*	54.26±12.06*
**Sex, M/F**	300 (168/132)	100(70/30)	300(229/71)
**Smoking(male)**	105(35%)	30(30%)	127(42.33%)
**Nonveg**	218 (72.66%)	74 (74%)	214(71.33%)
**Family History**	0	100 (100%)	20(6.66%)
**Total Cholesterol**	166.47±32.71	176.60±39.7*	247.91±39.8*
**Triglycerides(mg/dl)**	152.46±26.67	147.6±44.36	152.22±57.7
**HDL(mg/dl)**	44.72±11.047	35.5±12.12*	33.51±11.51*
**LDL(mg/dl)**	90.90±33.29	125±50.3*	174.62±37.7*
**VLDL(mg/dl)**	30.42±5.2	29.02±9.3*	30.62± 12.37

*Significant at p<0.05

### Analysis of Hs-CRP levels (ng/ml) in Coronary Artery Disease (CAD) patients, FDRS and Controls

**Fig 1:**
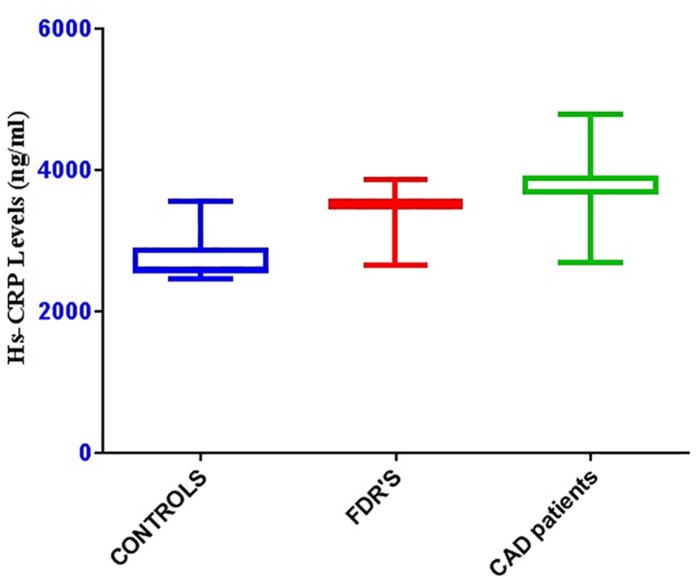
Analysis of Hs-CRP levels (ng/ml) in Coronary Artery Disease (CAD) patients, FDRS and Controls.

Hs-CRP levels were analyzed in coronary artery disease patients, FDRS and controls as shown in Fig. 1 and it has been found that the levels of CRP were significantly higher in CAD patients (4158. 82 ± 493.82), followed by FDRS (3603.79 ± 154.54) compared to controls (2626.39 ± 151.94).

### Genotypic distribution of IL-18 -137G/C Polymorphism in Coronary Artery Disease (CAD) patients, First Degree Relatives (FDRS) and Controls

No significant deviation from the Hardy Weinberg equilibrium was observed in controls, FDRS and in coronary artery disease patients. Genotype distribution, Allelic frequencies, Chi square, Odds ratio and 95% confidence interval (CI) of IL-18 –137G>C in controls, CAD patients and FDRS are presented in (Table 2) and it was found that homozygous GG is significantly associated with occurrence of coronary artery disease.

**Table 2:** Genotype distribution of IL 18–137 G/C polymorphism in Controls, CAD patients and FDRS (N = 700).

Model	Genotype	Controls N = 300 n (%)	CAD N = 300 n (%)	CAD Odds Ratio			FDRS N = 100 n (%)	FDRS Odds Ratio		
				χ2	Odds ratio (95% CI)	p-Value		χ2	Odds ratio(95% CI)	p-Value
**Co-dominant**	**GG**	176(58.7)	168(56)	0.001	1	0.99	65(65)	2.34	1	0.12
	**GC**	105(35)	102(34)		1.02 (0.72–1.44)		25(25)		0.64 (0.38–1.09)	
	**CC**	19(6.3)	30(10)	1.8	1.65 (0.90–3.05)	0.17	10(10)	2.38	1.43 (0.63–3.23)	0.12
**Dominant**	**GGGC-CC**	176(58.7) 124(41.3)	168(56) 132(44)	0.33	1.12 (0.81–1.54)	0.56	65(65) 35(35)	1	0.76 (0.48–1.22)	0.31
**Recessive**	**GG-GCCC**	281(93.7) 19(6.3)	270(90) 30(10)	2.22	1.64 (0.90–2.99)	0.13	90(90) 10(10)	1	1.64 (0.74–3.66)	0.31
**Overdominant**	**GG-CCGC**	195(65) 105(35)	198(66) 102(34)	0.02	0.96 (0.68–1.34)	0.86	75(75) 25(25)	2.9	0.62 (0.37–1.03)	0.08
**Log-Additive**	**- - - -**	**- - - -**	**- - - -**	**- - - -**	1.17 (0.91–1.50)	0.23	**- - - -**	**- - - -**	0.93 (0.65–1.34)	0.71
**Allele frequency**	**GC**	457(76) 143(24)	438(73) 162(27)	1.42	1.182 (0.91–1.53)	0.23	155(78) 45(22)	0.08	0.92 (0.6295–1.354)	0.77
**Minor allele frequency**	**C**	0.24	0.27	**- - - -**			0.22	**- - - -**		
**HWE (p-value)**		0.53	0.019	**- - - -**			0.007	**- - - -**		

### Hs-CRP levels (ng/ml) in association with IL-18 (-137 G/C) polymorphism in Coronary Artery Disease (CAD), First Degree Relatives (FDRS) and Controls

**Fig 2:**
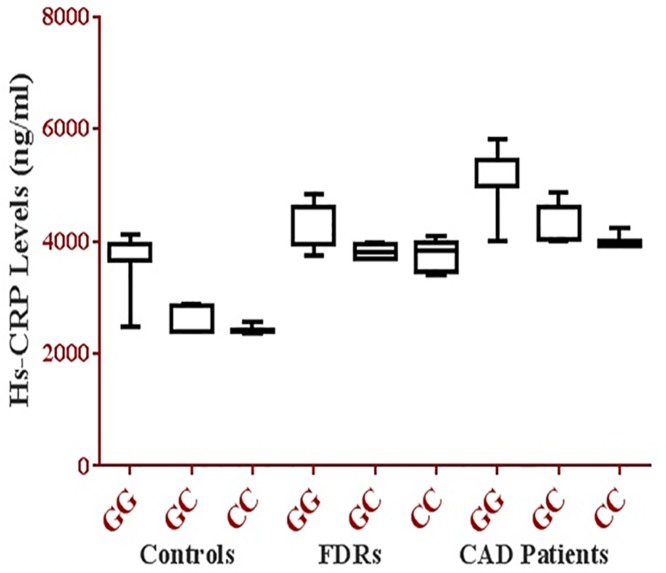
Hs-CRP levels (ng/ml) in association with IL-18 (-137 G/C) polymorphism in Coronary Artery Disease (CAD), First Degree Relatives (FDRS) and Controls.

Hs-CRP levels in association with IL-18 (-137 G/C) polymorphism in Coronary Artery Disease (CAD), First Degree Relatives (FDRS) and controls are presented in Fig. 2. The GG genotype was found to be associated with high Hs-CRP levels in coronary artery disease patients compared to GC Heterozygote and CC Homozygote. Study also revealed that the similar trend has been found in FDRS respectively.

It has been observed that there is no significant difference in the levels of Hs-CRP with IL 18 genotype of controls (Fig. 2) whereas the results were statistically significant at p< 0.05 between the 3 study groups.

### Analysis of Promoter binding site for IL -18-137G/C Polymorphism

**Fig 3:**
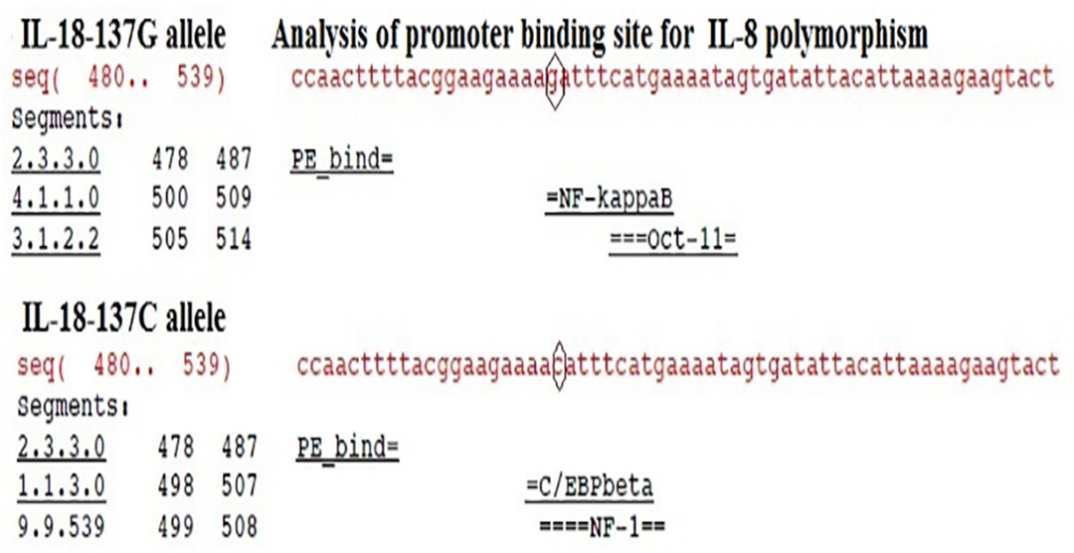
Analysis of Promoter binding site for IL -18-137 G/C Polymorphism

It has been found that IL-18 -137G allele has binding with NFkappaB whereas C allele has a additional binding site C/EBP beta and NF-1 (Fig. 3).

### Analysis of protein-protein interaction of IL-18

**Fig 4:**
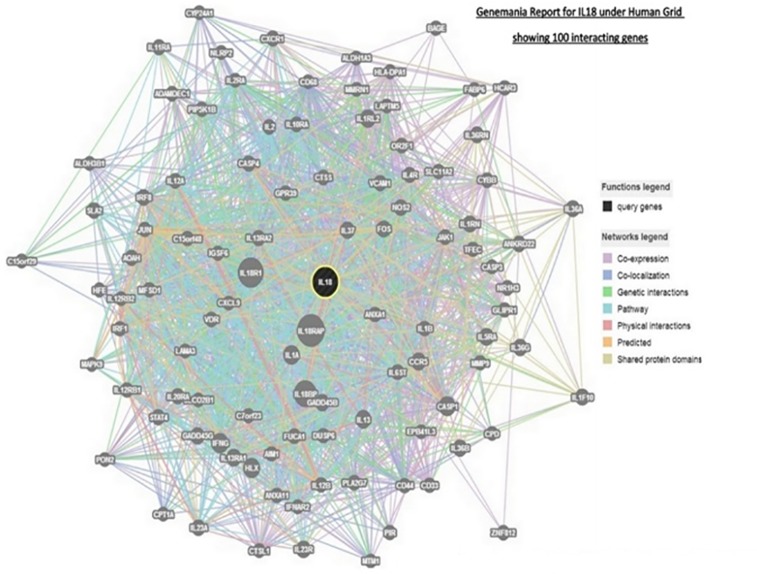
Analysis of protein-protein interaction of IL-18.

Of the 100 proteins interacting with IL18 in Genemania (Fig. 4), only 38 showed to share functional properties with IL18, of which the following are involved in causing inflammatory response- AOAH, CXCR1, IL18 RAP, IL13, CYBB, IL12B, ANXA1, IL1B, NR1H3, PLA2G7, IL2, IL6ST, IL23A, NOS2, FOS, IL1RN, CCR5 and IL1A.

Few like IFN-γ, IL12-β, IL2, IL12A, IL23A, IL23R and FOS are IL18 interactants and are also involved in IL17 regulation. IL-18 along with its interactants like IL12B, IL2, IL12A, IL23A and IL23R regulates natural killer cells involved in inflammation.

IL12RB2, NLRP2, IRF1, IL12B, IL12RB1, IL1B, IL2, IL12A, IL6ST, IL23A, IL23R, IFNG, IL1A and CXCL9 regulate the cytokine biosynthesis and activity. Cell adhesion is regulated by IL-18 and its interactants like ADAMDEC1, IL1RN, IL12B and IL1B.

The following 62 genes were found to interact with IL-18 however if their share any common function with IL18 is unknown: C7orf23, PON2, HFE, IGSF6, EPB41L3, LAMA3, LAPTM5, C15 or f 29, MMRN1, MTM1, CD68, CPD, IFNAR2, ALDH1A3, ALDH3B1, FABP6, IL2RA, CPT1A, SLCO2B1, CASP3, BAGE, CD44, SLC11A2, AIM1, MFSD1, CD33, MMP9, PIR, HCAR3, C15orf48, GLIPR1, GPR39, IL20RA, IL36RN, IL1RL2, IL13RA1, CASP1, IL18R1, IL5RA, IL37, IL36G, GADD45G, GADD45B, IL1F10, IL36A, IL36B, STAT4, IL4R, IL11RA, CASP4, IRF8, IL13RA2, ANKRD22, IL10RA, OR2F1, CTSS, FUCA1, ZNF812, PIP5K1B, ANXA11, TFEC and HLX.

AOAH, CTSL1, DUSP6, NLRP2, VCAM1, CYP24A1 and VDR co-express and co-localize with IL18. IL6ST only interacts with IL18 while IL13, IL2 and IL23R co-express and interact with IL18 through a common pathway. IFNG and IL18RAP follow the same pathway with IL18 and share physical interactions and so do IL1A in addition to co-expressing with it. AIM1, CPD, C7or f23, EPB41L3, GLIPR1, IFNAR2, IL20RA, IL23A, IRF8, JAK1, JUN, LAMA3, PLA2G7 and PON2 show genetic interactions though they do not directly interact with IL18. IL1A, IL1B and IL37 also show physical interactions with IL18 through intermediates. There are also predicted interactions between FOS, JUN, IL1A, IL1B, IL12A, 1L12B, IL12RB1, 1L23A, IFNG, IRF1, IRF8, CASP1, CASP3, CASP4, CXCL9, MAPK9, NOS2, STAT4 and IL18.

All the interactants some or the other way are involved in the regulation of immune response, activation and proliferation of T cells, leukocytes and lymphocytes, production of chemokines, cytokines and interferon-gamma.

## Discussion

Multiple interactions of several genetic, life style and environmental factors are involved in the complex pathophysiology of CAD. Some risk factors tend to be more prevalent within these families, including dyslipidemia, hypertension, diabetes, physical inactivity, high-fat dietary habits, smoking, excessive alcohol consumption, and obesity. It has been shown that individuals with a family history of CAD had significantly higher lipid levels (TC, TG, and LDL-C), glucose levels and had a higher BMI at young age [11]. There is also a tendency for similar socioeconomic status within families [12].

In the present study we have also observed significant association of lipid profiles (TC, TG, HDL, and LDL-C) and epidemiological characters (Age, Sex, Smoking, Non-veg) in First Degree Relatives (FDRS) and CAD patients.

Several reports revealed having a FDR with premature CAD is a significant autonomous risk factor for CAD development. It has been found that relativity of developing early-onset CAD in a first-degree relative is between 3.8 and 12.1 and depends on the age of onset (AOO) of the proband and higher risk correlates with earlier AOO.

It has also been found that FDRS have increased risk of death from cardiovascular disease compared to those without a family history [13]. The idea of inflammation that is associated with atherosclerosis has stimulated the study on inflammatory biomarkers for risk prediction of CAD.

Current studies have been steadily associated with several molecular proinflammatory biomarkers, markers of plaque destabilization and plaque rupture that can be used not only as prognosis for asymptomatic individuals for future CAD risk but also in patients with Acute Coronary Syndrome (ACS) apart from associated traditional risk factors [14].

CRP is a member of the pentraxin family and is most widely studied proinflammatory biomarker [14]. It has pleiotropic effects such as activation and chemoattraction of circulating monocytes, mediation of endothelial dysfunction, induction of a prothrombotic state, increase of cytokine release, activation of the complement system, and facilitation of extracellular matrix remodeling that may contribute to progression of vascular disease.

CRP is usually not seen in normal vessel wall and its presence and deposition early in atherosclerotic lesion is preceded by the monocytes appearance. It has been shown that, CRP can stimulate the expression of adhesion molecules and chemokines in human endothelial cells and acts in synergy with lipopolysaccharide to trigger endothelial cells to activate tissue factor production by monocytes. Thus, CRP is not only a marker of inflammation, but also an amplifier of it [15].

CRP and atherosclerosis had a link in the beginning as biomarker v/s mediator of atherosclerosis. This dogma has been revisited and suggested that CRP has a direct effect to promote atherosclerotic processes, contributing to the initiation and progression of atherosclerotic lesions.

Accumulating evidence suggests that circulating high-sensitivity CRP represents one of the strongest independent predictors of vascular death in a number of settings and it might be a strong biomarker than LDL cholesterol undoubtedly and gives a value to conventional Framingham risk assessment [16–19].

Previous data has shown that there is a strong relationship between Hs-CRP levels in myocardial infarction (AMI) [20] patients and unstable angina pectoris (UAP) patients [21]. It has also been demonstrated earlier that increased CRP levels among stable angina patients would confer greater risk of development of MI [22]. Studies have also shown an association of CRP levels with CAD risk factors like diabetes [23] and hypertension [24].

CRP gene polymorphisms have been previously shown to be linked to variation in concentration of CRP. It has been shown that different haplotypes of CRP gene have been associated with changes in the serum CRP levels [25,26].

In the present study we have also observed higher levels of Hs-CRP in CAD patients when compared to controls indicating the role of inflammation in CAD. So far, results from more than 25 different prospective studies demonstrated a significant and independent association between increased concentrations of CRP and future Cardio Vascular (CV) events.

Studies by Koenig on healthy middle-aged men indicate a strong link between CRP and risk of fatal or nonfatal CAD consequence [14]. Further, we have also evaluated the levels of Hs-CRP in FDRS and our results also suggest that CRP is one of the sensitive markers to identify inflammation and risk of future events of CAD in asymptomatic healthy individuals.

CRP may be involved in mechanism of atherosclerotic plaque development in part via IL18 activation. It has been suggested in recent studies that IL-18, a proinflammatory cytokine has an increasing evidence of being involved in both innate and acquired immune response and plays a key role in the inflammatory response that leads to atherosclerotic development.

The primary function of IL-18 is to stimulate T lymphocytes for the expression of IFN- γ which [27] takes part in recruitment of T cells and macrophages. It aids in the expression of MHC class II molecules and therefore results in the formation of foam cells.

Apart from this, it leads to increased activation of Anitgen Presenting Cells (APCs) and enhanced secretion of Th1-promoting cytokines [28]. Localized expression of higher IL-18 levels has also been associated with plaque instability in human atherosclerotic plaque [29]. Previous studies reported that IL-18 variants have been associated with varying expression of IL-18 and had an active role in clinical consequence CAD [30].

In addition, recent studies have interfaced IL-18 gene polymorphism with the CV mortality among CAD patients [29] and the consequence of MI among hypertensive patients and postmenopausal women [31].

IL-18 gene (-137G/C) promoter polymorphism has been associated with susceptibility to several diseases like CAD [32], Type 1 diabetes [33,34], rheumatoid arthritis [35,36], sarcoidosis [37], atopic eczema [38], adult-onset Still’s disease [26], and seasonal allergic rhinitis[24]. In contrary, no significant association was found when the IL18–137 G>C gene polymorphism was studied in patients with Crohn disease or ulcerative colitis [39].

It has been determined that SNPs in the promoter of the IL-18 gene at the position -137G/C (rs187238) can influence the expression of IL-18 and also potentially the expression of IFN-gamma [30]. Earlier studies by Giedraitis [40], Liang [41] have demonstrated that the change from guanine to cytosine affects the H4TF-1 (human histone H4 gene specific transcription factor-1)-binding site.

Therefore, the GG genotype of -137 polymorphism results in markedly higher transcription activity, leading to higher levels of the IL-18 protein than those made by the CC genotype and plays an important role in pathogenesis of CAD [30].

In the present study we have also found a significant increase in frequency of GG-genotype when compared to GC and CC genotypes in CAD patients & FDRS indicating the role of IL-18–137 G/C polymorphism in development of atherosclerosis and its complications.

Further we have also analyzed the results of Hs-CRP with IL-18 (-137 G/C) Polymorphism and found significantly higher levels of Hs-CRP in GG genotype when compared to GC & CC in CAD patients and FDRS respectively and there was no significant difference in controls.

Tojo et al, (2003) [42] demonstrated that CRP may contribute to the mechanism of endothelial inflammation in acute coronary syndrome by activation of the IL-18 system, which may amplify the inflammatory cascade in tissue injury in addition to initiating endothelial damage and atherogenesis promoted through the recruitment of leukocytes.

In addition to these studies we have also performed insilico analysis to predict transcriptional factors binding to IL-18 promoter region at -137G/C by using Alibaba 2.1 (Grabe 2002) online bio-informatics tool and found an additional transcription binding site for C/EBPbeta and NF-1 for C allele which may alter the levels of IL-18 protein [43].

We have also carried out the prediction of protein-protein interaction to find out the role IL-18 in relation to CAD and analysis revealed that IL-18 works by binding to the IL-18 receptor, and together with IL-12 induces cell-mediated immunity.

Stimulation of natural killer (NK) cells and certain T cells by IL-18 releases IFN-γ which plays a key role in activating the macrophages or other cells. Combined effect of IL-18 and IL-12 has been implicated in the suppression of IL-4 dependent IgE and IgG1 production, and enhanced IgG2a production in B cells. IL-18 binding protein (IL-18BP) can bind strictly to IL-18 and negatively regulate its biological activity. Being a pleiotropic cytokine IL-18 can also stimulate severe inflammatory reactions that establish its role in most inflammatory and autoimmune diseases.

Of the 38 genes annotated with known functions, only 14 seem to be closely related with IL-18, interacting directly with it in various ways leaving an impression that they might be playing a major role along with IL-18 in CAD. Establishing and reconfirming the similar relationship between these molecules in-vitro could open doors to drug discovery.

## Conclusion

In conclusion, the results of present study indicate the higher Hs-CRP levels and GG genotype of IL-18 (-137 G/C) polymorphism might help in risk prediction of CAD in healthy asymptomatic first degree relative (FDRS).
