# Correction: Evaluation of Hs-CRP Levels and Interleukin 18 (-137G/C) Promoter Polymorphism in Risk Prediction of Coronary Artery Disease in First Degree Relatives

**DOI:** 10.1371/journal.pone.0127609

**Published:** 2015-05-06

**Authors:** Rajesh Kumar G, Mrudula Spurthi K, Kishore Kumar G, Mohanalatha Kurapati, Saraswati M, Mohini Aiyengar T, Chiranjeevi P, Srilatha Reddy G, Nivas S, Kaushik P, Sanjib Sahu K, Surekha Rani H

In Table 2, there are errors in the “Dominant,” “Recessive,” “Overdominant,” and “Allele frequency” rows. Please view the corrected table below.

**Table 2:** Genotype distribution of IL 18–137 G/C polymorphism in Controls, CAD patients and FDRS (N = 700).

Model	Genotype	Controls N = 300 n (%)	CAD N = 300 n (%)	CAD Odds Ratio			FDRS N = 100 n (%)	FDRS Odds Ratio		
				χ2	Odds ratio (95% CI)	p-Value		χ2	Odds ratio(95% CI)	p-Value
**Co-dominant**	**GG**	176(58.7)	168(56)	0.001	1	0.99	65(65)	2.34	1	0.12
	**GC**	105(35)	102(34)		1.02 (0.72–1.44)		25(25)		0.64 (0.38–1.09)	
	**CC**	19(6.3)	30(10)	1.8	1.65 (0.90–3.05)	0.17	10(10)	2.38	1.43 (0.63–3.23)	0.12
**Dominant**	**GG**	176(58.7)	168(56)	0.33	1.12	0.56	65(65)	1	0.76	0.31
	**GC-CC**	124(41.3)	132(44)		(0.81–1.54)		35(35)		(0.48–1.22)	
**Recessive**	**GG-GC**	281(93.7)	270(90)	2.22	1.64	0.13	90(90)	1	1.64	0.31
	**CC**	19(6.3)	30(10)		(0.90–2.99)		10(10)		(0.74–3.66)	
**Overdominant**	**GG-CC**	195(65)	198(66)	0.02	0.96	0.86	75(75)	2.9	0.62	0.08
	**GC**	105(35)	102(34)		(0.68–1.34)		25(25)		(0.37–1.03)	
**Log-Additive**	- - - -	- - - -	- - - -	- - - -	1.17	0.23	- - - -	- - - -	0.93	0.71
					(0.91–1.50)				(0.65–1.34)	
**Allele frequency**	**G**	457(76)	438(73)	1.42	1.182	0.23	155(78)	0.08	0.92	0.77
	**C**	143(24)	162(27)		(0.91–1.53)		45(22)		(0.6295–1.354)	
**Minor allele frequency**	**C**	0.24	0.27	- - - -			0.22	- - - -		
**HWE (p-value)**		0.53	0.019	- - - -			0.007	- - - -		

Additionally, in Fig 3, the line “Analysis of promoter binding site for IL-8 polymorphism” is erroneously included. Please view the corrected figure here.

**Fig 3:**
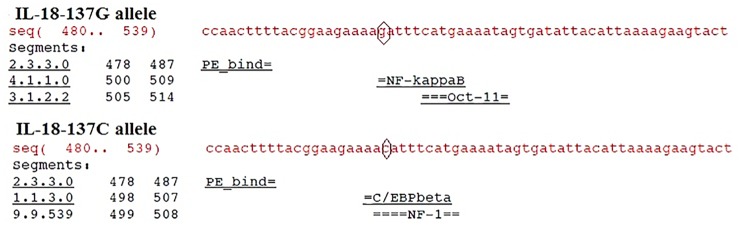
Analysis of Promoter binding site for IL -18-137 G/C Polymorphism.
